# Acute respiratory distress syndrome in COVID-19: possible mechanisms and therapeutic management

**DOI:** 10.1186/s41479-021-00092-9

**Published:** 2021-12-06

**Authors:** Anolin Aslan, Cynthia Aslan, Naime Majidi Zolbanin, Reza Jafari

**Affiliations:** 1 Department of Critical Care Nursing, School of Nursing and Midwifery, Tehran University of Medical Science, Tehran, Iran; 2 Immunology Research Center, Tabriz University of Medical Sciences, Tabriz, Iran; 3 Department of Immunology, Faculty of Medicine, Tabriz University of Medical Sciences, Tabriz, Iran; 4 Experimental and Applied Pharmaceutical Research Center, Urmia University of Medical Sciences, Urmia, Iran; 5 Department of Pharmacology and Toxicology, School of Pharmacy, Urmia University of Medical Sciences, Urmia, Iran; 6 Nephrology and Kidney Transplant Research Center, Clinical Research Institute, Urmia University of Medical Sciences, Shafa St., Ershad Blvd., P.O. Box: 1138, Urmia, 57147 Iran; 7 Hematology, Immune Cell Therapy, and Stem Cell Transplantation Research Center, Clinical Research Institute, Urmia University of Medical Sciences, Urmia, Iran

**Keywords:** COVID-19, Acute respiratory distress syndrome, SARS-CoV-2, Pandemics, ARDS

## Abstract

COVID-19 pandemic is a serious concern in the new era. Acute respiratory distress syndrome (ARDS), and lung failure are the main lung diseases in COVID-19 patients. Even though COVID-19 vaccinations are available now, there is still an urgent need to find potential treatments to ease the effects of COVID-19 on already sick patients. Multiple experimental drugs have been approved by the FDA with unknown efficacy and possible adverse effects. Probably the increasing number of studies worldwide examining the potential COVID-19 related therapies will help to identification of effective ARDS treatment. In this review article, we first provide a summary on immunopathology of ARDS next we will give an overview of management of patients with COVID-19 requiring intensive care unit (ICU), while focusing on the current treatment strategies being evaluated in the clinical trials in COVID-19-induced ARDS patients.

## Background

The 2019 novel coronavirus outbreak started in the Chinese city of Wuhan and quickly spread worldwide, leading the World Health Organization (WHO) to declare a Global Health Emergency on January 30, 2020 [1]. Later on, it was declared as a global pandemic on March 11 [2, 3]. This latest novel coronavirus, SARS-CoV-2, spreads more quick than its two closest common ancestors, the Severe acute respiratory syndrome coronavirus (SARS-CoV) and Middle East respiratory syndrome coronavirus (MERS-CoV), but has lower fatality. Infection can be spread through the droplet spray produced when coughing and sneezing by both symptomatic patients and asymptomatic carriers before the onset of the symptoms [4]. The respiratory system involvement is the most frequent complication of Coronavirus disease 2019 (COVID-19) [5]. Pneumonia caused by the SARS-CoV-2 virus presents with fever, dyspnea, and acute respiratory symptoms which can lead to refractory pulmonary failure [6, 7]. It is common among COVID-19 patients to develop acute respiratory distress syndrome (ARDS), a life-threatening form of respiratory failure [8, 9]. According to weighted averages calculated using data from individual studies which have data available for COVID-19, approximately 1/3 (33%) of hospitalized patients develop ARDS. And nearly 3/4 (75%) of COVID-19 patients admitted to the ICU have ARDS [8]. ARDS was initially defined in 1968 with clinical symptoms including acute hypoxemia, non-cardiac pulmonary edema, low pulmonary compliance, and increased work of breathing [10].

ARDS occurs due to both direct viral effects and host cell-derived substances [11]. Activated cells of the immune system release several products such as neutrophil myeloperoxidases and other proteinases, eosinophil major basic proteins and cationic proteins, and excessive production of proinflammatory cytokines including IL-6 and TNF-α, that can ensue aggravation of ARDS and extensive tissue damage resulting in multi-organ dysfunction and mortality [12, 13]. Although the exact mechanism of SARS-CoV-2 in ARDS is not fully understood yet, the induction of cytokine storm is considered to be one of the leading factors [14]. ARDS caused by COVID-19 differs considerably from ARDS caused by other factors based on Berlin criteria, and therefore treatment is different as well [5]. The onset time of COVID-19-associated ARDS is 8 to 12 days [15]. This is contrary to the Berlin ARDS criteria, which defined an onset limit of 1 week [16]. Patients with COVID-19 ARDS may have normal or even high lung compliance; this is not the case in patients with classic ARDS [5]. COVID-19 ARDS severity is redefined into three stages based on its specificity: mild, mild-moderate and moderate-severe [17]. Thus, the dependence on mechanical ventilation of COVID-19 ARDS is longer than that of non-COVID-19 ARDS [18]. In typical ARDS, the most frequently used adjuvant therapies are continuous neuromuscular blocking agents, high-dose corticosteroids, and recruitment maneuvers [19]. Due to the anti-inflammatory effects of corticosteroids, they are considered a possible treatment for ARDS and WHO strongly recommended systemic corticosteroid therapy for patients with severe and critical COVID-19, and recommended against corticosteroid therapy for patients with non-severe COVID-19 [20]. Several drugs including lopinavir-ritonavir, remdesivir, ruxolitinib and tocilizumab are undergoing clinical trials as a treatment for COVID-19, but yet no proven effective therapies currently exist [21–23].

In this review, we first describe the immunopathology of ARDS, then we intend to highlight the management of intensive care unit patients with COVID-19-related ARDS while focusing on the current status of promising emerging therapies although there is no particular recommended antiviral medication.

## Search strategy

We searched the PubMed and google scholar to retrieve eligible articles of any publication status and in English from December 1, 2019, to June 20, 2021, using the following key words: Therapeutic management, Therapeutic strategy, Antiviral therapies, Immunomodulatory therapies, anti-inflammatory drugs, NSAIDs, corticosteroids, MSC therapies, Interleukin-6 inhibitors, Janus kinase inhibitors, Convalescent plasma, IVIG, anti-fibrotic therapies, Anticoagulant therapies, Anti anaemic therapies. Each key word was searched with the following string of key words (using the “AND” operator): COVID-19-related-ARDS OR coronavirus-related-ARDS OR “SARS-CoV-2-related-ARDS” OR “Severe Acute Respiratory Syndrome Coronavirus 2” OR their derivates. In addition, we identified other eligible studies by searching the reference lists of relevant articles as well as unpublished studies in ClinicalTrials.gov. Studies which provided information on the treatment of COVID-19-related ARDS were identified. After screening title and abstracts for relevant studies, full texts were carefully checked for inclusion.

## Immunopathology of ARDS

It is discovered that for RNA viruses, coronavirus included, pathogen-associated molecular patterns (PAMPs) in the structure of viral RNA genomes, or the dsRNA replication intermediates, are recognized via either endosomal receptors, TLR3 (toll-like receptor 3) and TLR7 (toll-like receptor 7), or by cytosolic sensors of RNA, RIG-I/MDA (retinoic acid-inducible gene I/melanoma-differentiation-associated gene) [24]. These events lead to the transcriptional activation of interferon-stimulated genes and nuclear factor-κB-regulated genes (NF-κB) as well as immune effectors and regulatory cell recruitment [25]. Alveolar epithelial cells (AECs), alveolar macrophages (AMs), and dendritic cells (DCs) have key roles as sensor cells that detect danger signals via the receptors known as pattern recognition receptors (PRR) and initiate innate immunity. Recruitment of effector cells due to activated sensor cells lead to the secretion of a first wave of cytokines (AECs secrete IFNλ, CCL2, AMs secrete IFNα, IFNβ, IL-6, TNF, IL-12 and DCs secrete IL-12, IL-23, IL1β) to alert and stimulate resident lymphocytes [26]. The upregulation of type I IFN should be capable of suppressing the virus replication and dissemination during the early phase. In a SARS-CoV mouse model, it has been demonstrated that dysregulation of type I IFN and inflammatory monocyte macrophages cause fatal pneumonia. Consequently, exaggerated secretion of type I IFN and the infiltrated myeloid cells, negatively affect the result of the infection and are the key factors of lung damage and dysfunction [24]. Subsequently, as a part of the innate immunity the release of chemokines, including CCL2, CCL5, CXCL8, and CXCL10, results in the recruitment of neutrophils and NK cells to the lung parenchyma [25]. Neutrophils produce toxic agents, such as reactive oxygen species (ROS) and proteases. Considerable production of free radicals by neutrophils overwhelms endogenous anti-oxidant systems, leading to oxidative cell injury. This robust inflammatory reaction due to the activation of neutrophils plays a key role in the ARDS pathogenesis [27]. Similarly, NK cell recruitment during influenza virus infection exerts both pro-necrotic and -apoptotic effects *through the release of* granzymes and perforins; indeed, excessive NK cell–mediated cytotoxicity is linked to lethal influenza virus infection due to uncontrolled lung damage. Furthermore, infiltrating monocyte-derived macrophages and DCs by releasing other pro-inflammatory mediators such as TNF-α and nitric oxide, play key roles in influenza clearance and alveolar injury by inducing epithelial cell apoptosis [25]. DCs play important role in bridging the innate and adaptive immune systems via presenting pathogen antigens to the T cells located at lymph nodes. Once in the lung, cytotoxic T cells recognize the pathogen and kill pathogen-infected cells and remove the source of further infection. Helper T cells modulate inflammation in a myriad of strategies, including the excessive production of cytokines such as type-II interferon (IFN-II) and generate the memory against the pathogen and also activate B cells to differentiate in the germinal centers of conventional lymphoid tissues to produce antibodies specific to the pathogen [28]. Through the secretion of IL-10 and TGFβ, Treg cells suppress inflammation and restore homeostasis [26]. Comparing patients with ARDS, pneumonia, and healthy controls, researchers found that ARDS patients had increased IL-10-producing CD4^+^ T cells [29]. As well, ARDS survivors had more IL-10 producing CD4^+^ T cells than non-survivors [29]. In a small proportion of infected individuals, these immune processes can completely suppress viral replication or eliminate virus infection. Others have incomplete viral suppression and a reduction of circulating B and T cells followed by a mechanism as yet unknown. Cytokine storm, a serious condition caused by sustained viral replication, occurs in some patients and it has been shown to be the main cause of COVID-19 related ARDS [30]. Some of the cytokines and chemokines overexpressed during the cytokine storm include IL-1β, IL-2, IL-6, IL-10, TNF-α, IFN-γ, IP-10, MIP-1, and MIP-1α [31]. Serum IL-6 concentration has been associated with disease severity and mortality, suggesting IL-6 plays a central role [30]. As IL-6 circulates, it binds soluble IL-6 receptors, forming a complex with a gp130 dimer on the surface of some cells. The complex induces JAK-STAT3 activation in various cell types, including endothelial cells, leads to cytokine storm and finally may cause fatal symptoms such as ARDS in a subgroup of hospitalized COVID-19 patients [30, 32]. So blocking these immune pathways could be beneficial against cytokine storm and ARDS in patients suffering from COVID-19 in a severe form [30].

## Management of patients with ARDS in the intensive care unit (ICU)

A 1-week onset limit is defined by the ARDS Berlin criteria for a person to be diagnosed as having ARDS which is inconsistent with the onset time of COVID-19-related ARDS that is 8–12 days. So, greater attention must be paid to the ARDS development in patients with a course of longer than 1 week [5]. The management of ARDS patients is critical for survival, and should be taken into account by relevant specialists [33]. ARDS patients presenting with SpO_2_ < 94% on room air at sea level, respiratory rate > 30 breaths/min, PaO_2_/FiO_2_ < 300 mmHg or lung infiltrates > 50%, may require aerosol-generating procedures (AGPs). Compared to standard oxygen therapy, high-flow nasal oxygen (HFNO) decreases the requirement of endotracheal intubation in patients with ARDS. It has been suggested that HFNO may be safe in mild/moderate COVID-induced ARDS patients, and even in some moderate/severe patients [34]. Increasing lung capacity by the recruitment of previously collapsed units is often achieved by the use of high positive end-expiratory pressure (PEEP) levels, recruiting maneuvers, and prone positioning [35]. Prone positioning can improve oxygenation and survival in patients but care should be taken to turn them safely [36, 37]. For cases with suspected bacterial pneumonia or sepsis as a secondary infection, empiric antibiotics should be administered and re-evaluated daily, and, in case of no bacterial infection, antibiotic treatment should be de-escalated or stopped [38]. The evaluation should include the imaging of pulmonary (chest x-ray, ultrasound, and, if indicated, CT) and an electrocardiogram (ECG) if necessary and A laboratory test consisting of a complete blood count (CBC) with differential, metabolic panel, liver and renal function tests [38]. Even though measurement of inflammation markers such as CRP and D-dimer is not included in standard care, it can provide valuable prognostic information. Currently, limited information is available which suggests that the intensive care management of COVID-19 patients should be substantially different from the management of other ICU patients, although safety precautions are essential to avoid viral contamination. As for every ICU patient, successful COVID-19 clinical management depends on attention to the primary conditions leading to admission to ICU, but also to other comorbidities and hospital-acquired complications [38].

## Emerging therapies for COVID-19-induced ARDS

Although there is only one antiviral, Remdesivir, approved by the U.S. Food and Drug Administration (FDA) to treat hospitalized COVID-19 patients, many medications are being tested and currently, researchers are investigating other potential treatments for COVID-19 [38]. Two different strategies have been tested as potential “cures” for COVID-19, one strategy is to target the virus directly (reducing virus replication, receptor binding, etc.) and the other strategy is to modulate the innate and adaptive immune responses of the host against the virus infection (targeted or nonspecific immune-modulating drugs) [39]. A properly combined anti-inflammatory and anti-viral medication with doses adjusted according to the symptom severity and immune cell counts may help improve survival outcomes [28]. Much of the information available in research to date is based upon clinical trials, retrospective analyses, or uncontrolled case series, and so ultimate evidence of effectiveness for interventions is still required [39]. A list of drugs used for the treatment of COVID-19 patients is presented in Table 1 and a summary of the clinical trials investigating COVID-19 and COVID-19-associated ARDS management is provided in Table 2.

**Table 1:** Drugs used for COVID-19 respiratory distress

drug	Type of drug	mechanism of action	references
Remdesivir	antiviral drug	Inhibits SARS-CoV-2 RNA-dependent RNA polymerase	[40]
Favipiravir		targets RNA-dependent RNA polymerase enzymes	[41]
Lopinavir/Ritonavir		Inhibitors of the HIV protease	[42]
chloroquine and hydroxychloroquine		The precise mechanisms of action are unknown	[43]
Losartan		Angiotensin II receptor blocker, could prevent COVID-19 viral entry	[39]
dexamethasone	Immunomodulatory drug	Corticosteroid, suppress inflammatory reactions	[44]
methylprednisolone		Glucocorticoid, has anti-inflammatory and immunosuppressive effects	[45]
Tocilizumab		interleukin-6 receptor inhibitor, prevents the cytokine storm and dampens the inflammatory response	[46]
Baricitinib		JAK inhibitors, prevent the cytokine storm and dampen the inflammatory response	[47]
Ruxolitinib			[48]
heparin	Anticoagulant drug	binds to antithrombin III (AT-III)	[49]
Alteplase		Tissue plasminogen activator (tPA), breaks down blood clots	[50]
Pirfenidone	Anti-fibrotic drug	reduces fibroblast proliferation	[51]
Vadadustat	Anti anaemic drug	acts as a HIF prolyl-hydroxylase inhibitor	[52]

**Table 2:** Therapeutic clinical trials for COVID-19 and COVID-19-associated ARDS

NCT number	title	status	Condition or disease	Intervention/treatment	Phase	Sponsor
NCT04292730	Study to Evaluate the Safety and Antiviral Activity of Remdesivir (GS-5734™) in Participants With Moderate Coronavirus Disease (COVID-19) Compared to Standard of Care Treatment	Completed	COVID-19	Drug: Remdesivir Drug: Standard of Care	3	Gilead Sciences
NCT04610541	REMdesivir-HU Clinical Study and Severe Covid-19 Patients	Active, not recruiting	COVID-19	Drug: Remdesivir-HU	3	University of Pecs
NCT04600999	Clinical Trial of Favipiravir Treatment of Patients With COVID-19	Recruiting	COVID-19	Drug: Favipiravir	3	University of Pecs
NCT04276688	Lopinavir/ Ritonavir, Ribavirin and IFN-beta Combination for nCoV Treatment	Completed	COVID-19	Drug: Lopinavir/ritonavir Drug: Ribavirin Drug: Interferon Beta-1B	2	The University of Hong Kong
NCT04476992	Nitric Oxide Therapy for COVID-19 Patients With Oxygen Requirement (NICOR)	Active, not recruiting	Hypoxemia Pneumonia, COVID-19	Drug: Nitric Oxide-Sessions Drug: Nitric Oxide-Continuous and Sessions	1 2	Federal State Budgetary Scientific Institution, Research Institute of Cardiology
NCT04306393	Nitric Oxide Gas Inhalation in Severe Acute Respiratory Syndrome in COVID-19 (NOSARSCOVID)	Active, not recruiting	COVID-19	Drug: Nitric Oxide Gas	2	Massachusetts General Hospital
NCT04478071	Vadadustat for the Prevention and Treatment of Acute Respiratory Distress Syndrome (ARDS) in Hospitalized Patients With Coronavirus Disease 2019 (COVID-19)	Recruiting	Covid19 ARDS	Drug: vadadustat Drug: placebo	2	The University of Texas Health Science Center, Houston
NCT04653831	Treatment With Pirfenidone for COVID-19 Related Severe ARDS	Recruiting	Covid19 ARDS	Drug: Pirfenidone Other: Standard of care	N/A	Soroka University Medical Center
NCT04356833	Nebulised Rt-PA for ARDS Due to COVID-19 (PACA)	Recruiting	Covid19 ARDS	Drug: nebulised (rt-PA)	2	University College, London
NCT04453371	Impact of Tissue Plasminogen Activator (tPA) Treatment for an Atypical Acute Respiratory Distress Syndrome (COVID-19) (AtTAC)	Withdrawn	Covid19 ARDS	Drug: Tissue plasminogen activator Drug: Ringer solution	3	Negovsky Reanimatology Research Institute
NCT04357730	Fibrinolytic Therapy to Treat ARDS in the Setting of COVID-19 Infection	Active, not recruiting	Covid19 ARDS	Drug: Alteplase 50 MG [Activase]	2	Denver Health and Hospital Authority
NCT04530578	Severe Acute Respiratory Syndrome Respiratory Failure Acute Respiratory Distress Syndrome	Recruiting	Covid19 Pneumonia	Drug: Heparin sodium Drug: Enoxaparin	4	Clinica San Camilo, Argentina
NCT04350580	Polyvalent Immunoglobulin in COVID-19 Related ARds (ICAR)	Completed	Covid19 ARDS	Drug: Human immunoglobulin Drug: Placebo	3	Centre Hospitalier St Anne
NCT04261426	The Efficacy of Intravenous Immunoglobulin Therapy for Severe 2019-nCoV Infected Pneumonia	Not yet recruiting	Covid19	Drug: Intravenous Immunoglobulin Other: Standard care	2 3	Peking Union Medical College Hospital
NCT04380935	Effectiveness and Safety of Convalescent Plasma Therapy on COVID-19 Patients With Acute Respiratory Distress Syndrome	Recruiting	Covid19 ARDS	Biological: Convalescent plasma Drug: Standard of care	2 3	Indonesia University
NCT04382755	Zilucoplan® in Improving Oxygenation and Short- and Long-term Outcome of COVID-19 Patients With Acute Hypoxic Respiratory Failure (ZILU-COV)	Active, not recruiting	COVID-19	Drug: Zilucoplan® Drug: Placebo	2	University Hospital, Ghent
NCT04320277	Baricitinib in Symptomatic Patients Infected by COVID-19: an Open-label, Pilot Study. (BARI-COVID)	Not yet recruiting	Pharmacological Action	Drug: Baricitinib	2 3	Hospital of Prato
NCT04359290	Ruxolitinib for Treatment of Covid-19 Induced Lung Injury ARDS (RuXoCoil)	Active, not recruiting	Covid19 ARDS	Drug: Ruxolitinib administration	2	Philipps University Marburg Medical Center
NCT04322773	Anti-il6 Treatment of Serious COVID-19 Disease With Threatening Respiratory Failure (TOCIVID)	terminated (The study has been terminated due to changed clinical conditions and too few patients available)	Covid19	Drug: RoActemra iv Drug: RoActemra sc Drug: Kevzara sc Other: Standard medical care	2	Marius Henriksen
NCT04320615	A Study to Evaluate the Safety and Efficacy of Tocilizumab in Patients With Severe COVID-19 Pneumonia (COVACTA)	Completed	COVID-19 Pneumonia	Drug: Tocilizumab (TCZ) Drug: Placebo	3	Hoffmann-La Roche
NCT04466098	Multiple Dosing of Mesenchymal Stromal Cells in Patients With ARDS (COVID-19)	Active, not recruiting	Covid19 ARDS	Biological: Mesenchymal stromal cells Other: Placebo	2	Masonic Cancer Center, University of Minnesota
NCT04416139	Mesenchymal Stem Cell for Acute Respiratory Distress Syndrome Due for COVID-19 (COVID-19)	Recruiting	Covid19 ARDS	Biological: Infusion IV of Mesenchymal Stem cells	2	Instituto Nacional de Ciencias Medicas y Nutricion Salvador Zubiran
NCT04327401	COVID-19-associated ARDS Treated With Dexamethasone: Alliance Covid-19 Brasil III (CoDEX)	Terminated (The Data Monitoring Committee recommended to stop the trial based on the Recovery Trial results, which was accepted by the CoDEX Steering Committee.)	Covid19 ARDS	Drug: Dexamethasone	3	Hospital Sirio-Libanes
NCT04244591	Glucocorticoid Therapy for COVID-19 Critically Ill Patients With Severe Acute Respiratory Failure	Completed	Covid19	Drug: methylprednisolone therapy Other: Standard care	2 3	Peking Union Medical College Hospital
NCT04337190	Impact of Angiotensin II Receptor Blockers Treatment in Patients With COVID 19 (COVID-ARA2)	Recruiting	Covid19 ARDS	Biological: blood sampling	–	University Hospital, Angers
NCT04328012	COVID MED Trial - Comparison Of Therapeutics for Hospitalized Patients Infected With SARS-CoV-2 (COVIDMED)	Recruiting	Covid19	Drug: lopinavir/ritonavir Drug: Losartan Drug: Placebos	2 3	Bassett Healthcare
NCT04321096	The Impact of Camostat Mesilate on COVID-19 Infection (CamoCO-19)	Recruiting	Covid19	Drug: Camostat Mesilate Drug: Placebo oral tablet	1 2	University of Aarhus
NCT04328493	The Vietnam Chloroquine Treatment on COVID-19 (VICO)	Completed	Covid19	Drug: Chloroquine phosphate	2	Oxford University Clinical Research Unit, Vietnam
NCT04475991	Safety and Efficacy of Maraviroc and/or Favipiravir With Standard Therapy in Severe COVID-19 Adults (COMVIVIR)	Not yet recruiting	Covid19	Drug: Maraviroc + Currently used therapy Procedure: Curently used therapy for COVID-19 non-critical patients Drug: Favipiravir + Currently used therapy Drug: Maraviroc+Favipiravir+CT	2	Hospital General de México Dr. Eduardo Liceaga
NCT04307693	Comparison of Lopinavir/Ritonavir or Hydroxychloroquine in Patients With Mild Coronavirus Disease (COVID-19)	Terminated (Terminated early because no patients were further enrolled since mid-Apr 2020.)	Covid19	Drug: Lopinavir/ritonavir Drug: Hydroxychloroquine sulfate	2	Asan Medical Center

### Antiviral therapies /strategies

#### Remdesivir

Remdesivir is an antiviral drug that exhibits potent in vitro efficacy against SARS-CoV-2 [40]. Remdesivir is the first FDA approved medication (on October 22, 2020) for COVID-19 hospitalized patients. Remdesivir (also GS-5734) was developed by Gilead Sciences for the treatment of patients with Ebola virus disease in 2016 [53]. Although it has not been proved to be effective in human clinical trials for this disease, it has shown antiviral efficacy against coronaviruses including SARS-CoV-1 and MERS-CoV. Based on this information, remdesivir has earned notable attention for its likely use as an option for the treatment of SARS-CoV-2 [54, 55]. However, the evidence regarding the efficacy of remdesivir in treating COVID-19 is mixed.

In a randomised, double-blind, placebo-controlled, multicentre trial, Wang et al. demonstrated that remdesivir did not show statistically significant clinical improvements in hospitalized adults with severe COVID-19 [56]. The final results of a double-blind, randomized, placebo-controlled trial by Beigel et al. revealed that remdesivir is superior to placebo at reducing the duration of recovery in patients with lower respiratory tract infections who were hospitalized with Covid-19. According to their data, the remdesivir-treated patients showed a lower proportion of serious adverse events related to respiratory failure, indicating that remdesivir may have prevented the progression to more severe respiratory disease [57]. Based on a systematic review of RCTs and observational studies, Piscoya et al. investigated the effects of remdesivir in adult hospitalized patients with COVID-19 and evidence of respiratory insufficiency or pneumonia. The evidence was scarce on the efficacy and safety of 10-day remdesivir regimens, or when comparing 5-day or 10-day regimens to standard of care [55]. Regardless of the FDA approval, several ongoing RCTs need to be completed in order to evaluate if remdesivir has a clinically effectiveness and safety profile. Emdesivir cannot be concluded to be effective for treating COVID-19 until stronger evidence is available [55].

#### Favipiravir

Favipiravir is a viral RNA polymerase inhibitor, already demonstrated *activity against* influenza A and B [41]. The clinical trials of Favipiravir generally do not include patients with critical or severe conditions [58]. However, these case reports suggest that favipiravir is effective in treating patients with severe or critical conditions. Takahashia et al. shared their experiences with three COVID-19 patients: two were in critical condition, and one was in a very severe condition. All three cases required high dose oxygen therapy or ECMO. Favipiravir helped them recover from SARS-CoV-2 pneumonia, and the oxygenation treatment was tapered. According to their report of three COVID-19 cases, favipiravir may be effective in preventing pneumonia progression and cytokine production, improving respiratory function, and producing immediate effects, even in serious or critical conditions [58].

#### Lopinavir/ritonavir

Lopinavir/Ritonavir are inhibitors of the HIV protease and are routinely used in the treatment of AIDS [42]. In a randomized, controlled, open-label trial, Cao et al. observed that lopinavir-ritonavir therapy compared with standard care, in adult patients with severe Covid-19, did not yield any positive results. They found that Lopinavir/ritonavir treatment failed to reduce risk of death, improve clinical outcomes, or decrease throat viral RNA detectability in patients with severe Covid-19. The results of future trials in patients with severe COVID-19 may confirm or exclude the possibility that the treatment is beneficial [42].

#### Chloroquine and hydroxychloroquine (Clq/HClq)

ACE2 allows SARS-CoV-2 virus to enter cells, disrupting the renin-angiotensin-aldosterone axis and possibly contributing to lung damage [59]. The antimalarial medications, chloroquine, and hydroxychloroquine interrupt ACE2 binding and block viral entry, moreover, they also could affect endosomal and lysosomal pH, which can suppress the merging of the virus with the host cells [60]. These medications also inhibit the secretion of pro-inflammatory cytokines [43]. Chloroquine has especially been reported to suppress lung injury induced by influenza A H5N1 in preclinical designs [61]. In an open-label RCT, as a result of the addition of Clq/HClq to standard care, patients admitted with severe COVID-19 significantly worsened in their clinical status, were at greater risk of renal dysfunction, and required more IMV, even though mortality did not differ. This study concludes that Clq/HClq should be avoided in patients with a more severe form of COVID-19 pneumonia, and can be used to inform clinical practice and guidelines [62]. Furthermore, according to a meta-analysis, treatment with Clq/HClq does not result in any benefit in mild, moderate, or severe COVID-19 patients. When comparing treatment groups and controls in pooled analysis, no significant difference was observed in clinical recovery, viral clearance, and length of hospital stay. Based on the currently available RCTs, Clq/HClq has no added benefit in the treatment of COVID-19 patients [63]. Since the HCQ trial failed to show any benefit, WHO and the National Institute of Health discontinued the trial for hospitalized COVID patients [64].

#### Losartan

Angiotensin receptor blockers (ARBs), such as losartan, may also alleviate some of adverse effects of ACE2 induction [59]. Losartan is currently being tested in patients with COVID-19 [39, 65]. Among patients with COVID-19, hypertension (HTN) is a major cause of acute respiratory failure, hospitalization, and mortality [66]. Losartan and amlodipine were compared in patients with primary HTN and COVID-19 by Nouri-Vaskeh et al. in a randomised clinical trial. Losartan or amlodipine administration did not appear to have any priority for COVID-19 patients with primary HTN [66]. Further, Bolotova et al. in a feasibility study noted that losartan was well tolerated among hospitalized COVID-19 patients with HTN and did not worsen symptoms [67]. Bengtsone et al. examined the safety of using losartan to treat COVID-19-related respiratory failure in an open-label, non-randomized trial using an external, post-hoc control group. The study found that Losartan was safe for acute respiratory compromise caused by COVID-19. However, randomized trials are required to evaluate true efficacy [68].

### Immunomodulatory therapies

The selection of anti-inflammatory drugs and their therapeutic doses ought to be based on the severity of the symptoms and should be monitored with the number of immune cells present in the complete blood count (CBC) [69]. Management of COVID-19 inflammation with NSAIDs and systemic corticosteroids has been controversial [70]. As overt symptoms of the cytokine storm emerge, anti-inflammatory cytokines like Interferon beta-1b (IFN-β-1b), and therapeutic antibodies targeting pro-inflammatory cytokines or their signaling pathways like Tocilizumab (anti-IL-6), Adalimumab (anti-TNFα), Anakinra (anti-IL-1), and Baricitinib (Janus kinase inhibitor) might be beneficial [71, 72].

#### Corticosteroids

In consideration of the cytokine storm observed during SARS-CoV, MERS-CoV, and SARS-CoV-2 infections, corticosteroids have been commonly used to treat serious illness, for the possible recovery of lung injury induced by inflammation [73]. Despite the adverse effects of corticosteroid use, such as delayed viral clearance and opportunistic infections, and while initially WHO recommended against corticosteroid therapy, as of September 2, 2020, WHO strongly recommended systemic corticosteroid therapy for patients with severe and critical COVID-19 rather than no systemic corticosteroids [20, 74]. The choice of corticosteroid therapy and the length of treatment can be significant and must be taken into notice [75]. Several clinical trials evaluated the effectiveness of corticosteroid and glucocorticoid therapy in critically ill patients with COVID-19 [45, 76]. In a multicentre, randomised controlled trial done by Villar et al. high doses of dexamethasone, 20 mg per day, from day 1 to 5, then 10 mg per day from day 6 to 10 were administered in all stages of ARDS, even in the mild cases. They demonstrated that early treatment of dexamethasone could decrease overall mortality and ventilator duration in patients with established moderate-to-severe ARDS [44]. Furthermore, in a randomized clinical trial, Tomazini et al. evaluated the efficacy of i.v. dexamethasone administration in COVID-19 induced moderate to severe ARDS patients. In these patients, standard treatment plus i.v. dexamethasone led to a statistically significant increase in days alive and free of mechanical ventilation over 28 days compared with standard treatment alone [77]. Moreover, in a controlled, open-label trial, it has been determined that dexamethasone use reduced 28-day mortality in hospitalized Covid-19 patients’ who received either mechanical ventilation or oxygen, but not those receiving no respiratory support [78]. Some preliminary trial results suggest methylprednisolone and dexamethasone can be used for the severe form of COVID-19 [79]. Using a randomized control study, Ranjbar et al. assessed the efficacy of methylprednisolone in hospitalized COVID-19 patients, comparing it to regular dexamethasone treatment. Compared to the use of 6 mg/day of dexamethasone in patients admitted to hospital with COVID-19 pneumonia, the administration of 2 mg/kg per day of intravenous methylprednisolone resulted in a shorter hospital stay and less need for mechanical ventilation. Methylprednisolone demonstrated better outcomes in COVID-19 hypoxic patients when compared to dexamethasone [80]. The results of a meta-analysis of seven RCTs and 6250 severe COVID-19 cases indicated that corticosteroid therapy reduced all-cause death and disease progression rather than increasing adverse events [81]. In a systematic review and meta-analysis, Chaudhuri et al. summarized RCT findings concerning corticosteroids’ role in ARDS of any cause. Corticosteroids were proposed to be beneficial for patients with all forms of ARDS regardless of their causes. Corticosteroids might reduce mortality rates in ARDS patients and the need for mechanical ventilation. COVID-19 and non-COVID-19 ARDS patients showed the same effect across different corticosteroid types and dosages [82]. Kumakawa et al. reported a patient with severe ARDS caused by COVID-19. Treatment in this 67-year-old man was managed with late administration of i.v. steroids from day 20th of administration until 27th which was successful. The current report highlights the need for future trials to evaluate the best treatment timing and doses for ARDS induced by COVID-19, as well as selecting the optimal population for different severity COVID-19-induced ARDS [83]. Due to the affordability and accessibility of corticosteroids in healthcare systems trembling under the strain of the worldwide outbreak of this coronavirus, this area of research should be a universal priority [74].

#### Mesenchymal stem cell (MSC) therapies

Mesenchymal stem cells have exhibited immunoregulatory capability which can suppress inflammatory reactions. Chan et al. reported that MSC therapy has beneficial effects on H5N1- induced acute lung injury and may be beneficial to patients with a severe pulmonary illness caused by influenza viruses such as H5N1 and H7N9 [84].

Lanzoni et al., in a double-blind RCT, examined the safety and efficacy of allogenic UC-MSC infusions in patients with ARDS associated with COVID-19. Their trial results revealed that UC-MSC infusions are safe for COVID-19 patients with ARDS. Furthermore, compared to controls, UC-MSC treatment reduced SAEs, mortality, and recovery time [85]. Moreover, Shi et al. conducted a double-blind, placebo-RCT at two medical centers in Wuhan, China, assessing the safety and efficacy of iv administration of UC-MSCs in COVID-19 patients with severe lung damage. Following administration of UC-MSC, the lesions of the lung solid component were resolved faster as well as the capability of the integrated reserve improved [86]. Liang et al. noted that the optional transfer of hUCMSCs, combined with other therapeutics presented good clinical outcomes for a severely ill patient with COVID-19 and acute lung inflammation. Albeit only a case was designated there, it would further be especially valuable to inspire more clinical investigation to manage likewise critically ill patients with COVID-19 [87]. Moreover, Leng et al. [80] reported a single-dose clinical trial of MSC therapy in 7 patients with COVID-19 induced ARDS and 3 controls, and a case study by Liang et al. [81] reported the resolution of all COVID-19 manifestations in a severely ill woman on a ventilator who was administered three intravenous MSC doses. In these 2 reports, all of the 8 patients with COVID-19 induced ARDS made a good recovery following the administration of MSCs, although the follow-up periods differed, and there was no stability in the measurement of biological variables [88]. MSCs have been shown to be effective and safe in preclinical studies of ARDS models, and the results from Covid-19 clinical trials demonstrated their potential efficacy. Nevertheless, more large-scale trials are needed to verify MSC’s efficacy, particularly in patients with ARDS who have been diagnosed with Covid-19. Additionally, research is needed to establish the optimal cell source, dose and route of MSCs therapies, to develop a safe and effective treatment option for ARDS patients, particularly those who suffer from Covid-19 [89].

#### Interleukin-6 inhibitors

Excessive secretion of IL-6 can cause an acute systemic inflammation referred to as cytokine release syndrome (CRS). In the pathogenesis of COVID-19 pneumonia, it has been found that a CRS involving a considerable proinflammatory cytokine secretion occurred, including IL-6, IL-1, and TNF-α [90]. Xu et al. in a non-controlled retrospective study of 21 patients with SARS-CoV-2-induced ARDS demonstrated that treatment with tocilizumab (TCZ), an interleukin-6 receptor antagonist, could decrease the number of white blood cells and improve CT lung opacity and lung oxygenation [46]. Based on this data, on March 3, 2020, TCZ, an anti-IL-6 receptor monoclonal antibody, was included in the 7th edition of COVID-19 therapy recommendations by China’s National Health Commission (NHC) [90]. A single center-based study with 100 patients in Brescia, Italy evaluated the efficacy of intravenous administration of TCZ in the treatment of severe COVID-19 pneumonia and ARDS patients. They observed that more than three-quarters of patients displayed improvements in their clinical outcomes [91, 92]. In a RCT of hospitalized patients with COVID-19 pneumonia and PaO2/FIO2 ranging from 200 to 300 mmHg, no difference was shown in the progression of disease between the tocilizumab and the standard care group [93]. A systematic review and meta-analysis was conducted by Pinzon et al. to evaluate evidence regarding the effectiveness of IL-6 inhibitors in the treatment of COVID-19. In patients with COVID-19, IL-6 inhibitors have shown to be beneficial in reducing mortality, particularly in severely ill cases [94]. However, additional blinded, placebo-controlled, RCTs should be conducted in well-established settings to evaluate the risks and benefits of IL-6 inhibitor agents across the disease spectrum [93].

#### Janus kinase inhibitors (e.g., baricitinib, Ruxolitinib)

Another drug that can be used to block viral entry through ACE2-mediated virus endocytosis is baricitinib, an inhibitor of JAK, that also prevents the cytokine storm and dampens the inflammatory response [47]. One meta-analysis by Chen et al. evaluated 11 studies of the safety and effectiveness of ruxolitinib and baricitinib in COVID-19 patients. They found these drugs decreased the use of IMV, had borderline effects on rates of ICU admission and ARDS and did not decrease interval of hospitalization. Among the treatments, baricitinib showed the most convincing reduction in the risk of death [95]. Capochiani et al. investigated the use of JAK inhibitors in the management of patients infected with SARS-CoV-2, including patient selection and dosing and administration information. Despite the small number of patients collected, they reported encouraging results regarding using ruxolitinib as a possible treatment option for severely ill patients with COVID-19 who develop respiratory distress [96]. In another study, Neubauer et al. have described the first successfully treated case with COVID-19-related ARDS using ruxolitinib. They observed that ruxolitinib therapy resulted in a decreased ARDS-associated inflammatory cytokines levels such as IL-6 and the acute phase protein ferritin, and also was associated with a rapid improvement in cardiac and respiratory systems [48]. Currently, clinical trials are ongoing to study both ruxolitinib and baricitinib in a prospective manner [48, 74, 97].

#### Convalescent plasma

Convalescent plasma treatment is nothing new; physicians have used it for SARS, pandemic 2009 influenza A (H1N1), avian influenza A (H5N1), several hemorrhagic fevers including Ebola, and other viral infections [98]. Convalescent plasma from the blood of people who’ve recovered contains neutralizing antibodies against viral proteins in almost all patients with COVID-19 [99, 100]. Therefore, it might be worthwhile to investigate the safety and effectiveness of convalescent plasma therapy in COVID-19 cases [101]. A randomized controlled trial by Pouladzadeh et al. found that CP had remarkable immunomodulatory and antiviral properties, reducing the cytokine storm and improving the clinical scores in COVID-19 patients, but had little impact on mortality [102].. Furthermore, Raymond et al. in a randomized, parallel arm, phase II trial with patients with severe COVID-19 disease and mild to moderate ARDS investigated the clinical and immunological benefits of convalescent plasma transfusion. The convalescent plasma treatment arm did not demonstrate statistically significant differences in clinical outcomes across all age groups, though patients with severe ARDS aged less than 67 years were found to experience immediate hypoxia reduction, shorter hospital stays, and improved survival. This study suggested that a precise targeting of severe COVID-19 patients is necessary to achieve efficacy [103]. In a study by Shen et al., 5 critically ill patients with laboratory-confirmed COVID-19 and ARDS were treated with convalescent plasma. As assessed by Ct, treatment with convalescent plasma leads to a decline in viral load within days, and clinical improvement of patients, as indicated by body temperature reduction, improvement in P/F ratio, and chest imaging. By 9 days after plasma administration, four patients who had been receiving extracorporeal membrane oxygenation (ECMO) and mechanical ventilation no longer required breathing support [104]. Moreover, According to Allahyari et al., early administration of CP can help defuse the symptoms of severe COVID-19 patients with mild or moderate ARDS, who are at risk of progressing to critical state [105].

#### Intravenous immune globulin (IVIG)

Intravenous immunoglobulin (IVIG) has been investigated as an alternative immune-modulator, and IVIG therapy in a high dose has shown useful effects for immune-mediated diseases, such as Kawasaki disease and other diseases [106]. However, the exact mechanisms of action of IVIG in immune-mediated diseases are still unknown, but IVIG may act on the host’s hyperimmune reactions by binding to immune cell receptors, etiologic substances including pathogenic proteins (PPs), or other proteins that are linked to inflammation [106]. A double-blind, randomized clinical trial demonstrated that the administration of IVIg to patients with severe COVID-19 infection who failed to respond to initial treatment has significantly improved their clinical outcome. Still, several multicenter trials with larger sample sizes must be conducted to provide more information regarding the drug’s suitability as a standard treatment [107]. In recent case reports, IVIG therapy has proved beneficial for patients with COVID-19, including three cases of ARDS. In a retrospective study, early IVIG treatment of COVID-19-related ARDS resulted in a smaller mortality and shorter ventilator time [108]. With regards to IVIg’s high price, it is recommended that it should be considered for patients with > 30% lung involvement in lung CT scans, persistent dyspnea, persistent satO2 under 90%, and individuals with progressive lung involvement in serial lung CT scans, especially in younger cases [107].

### Anticoagulant and anti-fibrotic therapies

#### Heparin

Disordered coagulation, specifically, pulmonary microvascular thrombosis is increasingly in association with the pathogenesis of severe COVID-19 respiratory failure. Treatment with anticoagulants, mainly low molecular weight heparin (LMWH), has also been found to be associated with better treatment outcomes in severe coronavirus patients with evidence of activation of the coagulation system such as markedly elevated D-dimer levels [49]. A prophylactic dose of LMWH may be recommended for hospitalized patients. LMWH also exerts anti-inflammatory effects that might confer protection [109]. Currently, a clinical trial is ongoing to test its potential to reduce mortality in patients with Severe COVID-19 [110].

#### Alteplase

It has been suggested that targeting coagulation and fibrinolysis could improve the clinical outcomes of ARDS patients [111]. In particular, plasminogen activators have received strong support to limit the progression of ARDS and reduce ARDS-induced death from animal models [112–114] and a phase 1 human clinical trial [115]. According to Wang et al., three COVID-19 patients with ARDS being ventilated and being treated with tPA (Alteplase) showed temporally improved respiratory status, with one of them demonstrating a durable response [116]. Moore et al. have suggested that Alteplase administration can be used as compassionate salvage therapy in patients with COVID-19 related ARDS, but details remain to be determined about the dose, administration routes, and duration of treatment [50]. At the moment, clinical trials are in progress to evaluate the effects of tPA on improving the respiratory function of ARDS patients [117–119].

#### Pirfenidone

Pirfenidone which is approved for the treatment of mild to moderate idiopathic pulmonary fibrosis (IPF), may prevent the invasion and cytokine storm of COVID-19 in pneumocytes and other tissues by inhibiting apoptosis, decreasing expression of ACE receptors, suppressing inflammation through various mechanisms, and reducing oxidative stress [120]. Consequently, it can be effective against severe viral inflammation, ARDS, and ARDS fibrosis [51]. A new clinical trial aims to determine the safety and efficacy of treatment with Pirfenidone versus standard of care (SoC) in COVID-19 patients with severe ARDS (NCT04653831).

### Anti anaemic drugs

#### Vadadustat

Vadadustat acts as a hypoxia-inducible factor prolyl hydroxylase inhibitor (HIF-PHI) and can trigger the body’s protective response to oxygen deficiency [52]. Alveolar inflammation can be dampened by stabilizing HIF, which is one of the main challenges that patients with COVID-19-related lung disease face when they develop ARDS [121]. The efficacy of vadadustat for ARDS prevention and treatment is going to be examined in a new clinical trial in hospitalized patients with COVID-19 [18].

### Nitric oxide (NO)

During the SARS-CoV outbreak, in 2004, a pilot study revealed that low dose NO (max 30 ppm) inhalation could shorten the duration of the ventilator for patients diagnosed with SARS-CoV infection [122]. Furthermore, strong evidence suggests that inhaled NO can decrease inflammatory cell-mediated lung damage via suppressing activation of neutrophils and subsequent secretion of pro-inflammatory cytokines [123]. Although there is no epidemiological evidence supporting the use of inhaled NO to improve outcomes for COVID-19 patients, because of the genetic similarities between the two viruses, similar therapeutic effects of NO could be expected for COVID-19 patients [124]. Based on this experience, several medical institutes have begun clinical trials [125], and now a phase 2 clinical study of inhaled NO is being performed for COVID-19 patients who required mechanical ventilation for ARDS to confirm whether inhaled NO could be a life-saving intervention for managing COVID-19 ARDS [126, 127].

## Conclusion

2020 has been a challenging year for all with the COVID-19 pandemic as a live issue affecting people globally. Although 58 COVID-19 vaccines have been developed in clinical trials by several manufacturers, with some vaccines proving to be over 90% effective in preventing the disease in clinical trials [128], But it might take years for enough coverage to create herd immunity, and vaccine escape mutants are a threat to this progress [129]. So, there is still an urgent need to develop targeted therapies to combat COVID-19 and its complications [23]. In this sense, it is hoped that extensive research worldwide into possible therapies for patients suffering from COVID-19 will result in the rapid identification of effective ARDS treatments.

## Data Availability

Not applicable.

## Abbreviations

AECs: Alveolar epithelial cells
AMs: Alveolar macrophages
ARDS: Acute Respiratory Distress Syndrome
CBC: Complete blood count
COVID-19: Coronavirus disease 2019
CRS: Cytokine release syndrome
DCs: Dendritic cells
ECG: Electrocardiogram
FDA: Food and Drug Administration
HFNO: High-flow nasal oxygen
ICU: Intensive care unit
IFN-II: Type-II interferon
IVIG: Intravenous immune globulin
JAK: Janus kinase
MERS-CoV: Middle East respiratory syndrome coronavirus
MSC: Mesenchymal Stem Cell
NF-κB: Nuclear factor-κB-regulated genes
NHC: National Health Commission
NO: Nitric oxide
PEEP: Positive end-expiratory pressure
PRRs: Pattern recognition receptors
RIG-I/MDA: Retinoic acid-inducible gene I/melanoma-differentiation-associated gene
ROS: Reactive oxygen species
SARS-CoV: Severe acute respiratory syndrome coronavirus
TLR: Toll-like receptor
tPA: Tissue plasminogen activator
WHO: World Health Organization

## References

[1] Wee SL, McNeil DG, Hernández JC. WHO declares global emergency as Wuhan coronavirus spreads. New York Times. 2020;30.

[2] Colavita F, Lapa D, Carletti F, Lalle E, Bordi L, Marsella P, Nicastri E, Bevilacqua N, Giancola ML, Corpolongo A, Ippolito G. SARS-CoV-2 isolation from ocular secretions of a patient with COVID-19 in Italy with prolonged viral RNA detection. Ann Intern Med. 2020;173(3):242–3. 10.7326/M20-1176 PMC7175424 32302380

[3] Hu Y, Sun J, Dai Z, Deng H, Li X, Huang Q, Wu Y, Sun L, Xu Y. Prevalence and severity of corona virus disease 2019 (COVID-19): A systematic review and meta-analysis. J Clin Virol. 2020;127:104371. 10.1016/j.jcv.2020.104371 PMC7195434 32315817

[4] Singhal T. A review of coronavirus disease-2019 (COVID-19). Indian J Pediatr. 2020;87(4):281–6. 10.1007/s12098-020-03263-6 PMC7090728 32166607

[5] Li X, Ma X (2020). Acute respiratory failure in COVID-19: is it “typical” ARDS?. Crit Care.

[6] Ghelichkhani P, Esmaeili M. Prone position in management of COVID-19 patients; a commentary. Arc Acad Emerg Med. 2020;8(1). PMC7158870 32309812

[7] Henry BM, Lippi G. Poor survival with extracorporeal membrane oxygenation in acute respiratory distress syndrome (ARDS) due to coronavirus disease 2019 (COVID-19): pooled analysis of early reports. J Crit Care. 2020;58:27. 10.1016/j.jcrc.2020.03.011 PMC7118619 32279018

[8] Tzotzos SJ, Fischer B, Fischer H, Zeitlinger M (2020). Incidence of ARDS and outcomes in hospitalized patients with COVID-19: a global literature survey. Crit Care.

[9] Wu C, Chen X, Cai Y, Zhou X, Xu S, Huang H (2020). Risk factors associated with acute respiratory distress syndrome and death in patients with coronavirus disease 2019 pneumonia in Wuhan, China. JAMA Intern Med.

[10] Matthay MA, Zemans RL, Zimmerman GA, Arabi YM, Beitler JR, Mercat A (2019). Acute respiratory distress syndrome. Nat Rev Dis Primers.

[11] Chaithanyaa N, Devireddy SK, Kumar RK, Gali RS, Aneja V (2012). Sympathetic ophthalmia: a review of literature. Oral Surg Oral Med Oral Pathol Oral Radiol.

[12] Lee K-Y, Rhim J-W, Kang J-H (2011). Hyperactive immune cells (T cells) may be responsible for acute lung injury in influenza virus infections: a need for early immune-modulators for severe cases. Med Hypotheses.

[13] Lee K-Y (2015). A common immunopathogenesis mechanism for infectious diseases: the protein-homeostasis-system hypothesis. Infect Chemother.

[14] Ragab D, Salah Eldin H, Taeimah M, Khattab R, Salem R (2020). The COVID-19 cytokine storm; what we know so far. Front Immunol.

[15] Huang C, Wang Y, Li X, Ren L, Zhao J, Hu Y (2020). Clinical features of patients infected with 2019 novel coronavirus in Wuhan, China. Lancet.

[16] Force ADT, Ranieri V, Rubenfeld G, Thompson B, Ferguson N, Caldwell E (2012). Acute respiratory distress syndrome. Jama..

[17] Zheng R, Hu M, Li R (2020). Respiratory treatment procedures in patients with severe novel coronavirus infected pneumonia: an expert opinion. Chin J Crit Care Intensive Care Med.

[18] Bain W, Yang H, Shah FA, Suber T, Drohan C, Al-Yousif N (2021). COVID-19 versus non–COVID-19 acute respiratory distress syndrome: comparison of demographics, physiologic parameters, inflammatory biomarkers, and clinical outcomes. Ann Am Thoracic Soc.

[19] Song F, Shi N, Shan F, Zhang Z, Shen J, Lu H (2020). Emerging 2019 novel coronavirus (2019-nCoV) pneumonia. Radiology..

[20] Organization WH. Corticosteroids for COVID-19: living guidance, 2 September 2020: World Health Organization; 2020.

[21] Zhou F, Yu T, Du R, Fan G, Liu Y, Liu Z, Xiang J, Wang Y, Song B, Gu X, Guan L. Clinical course and risk factors for mortality of adult inpatients with COVID-19 in Wuhan, China: a retrospective cohort study. Lancet. 2020;395(10229):1054–62. 10.1016/S0140-6736(20)30566-3 PMC7270627 32171076

[22] Gautret P, Lagier JC, Parola P, Meddeb L, Mailhe M, Doudier B, Courjon J, Giordanengo V, Vieira VE, Dupont HT, Honoré S. Hydroxychloroquine and azithromycin as a treatment of COVID-19: results of an open-label non-randomized clinical trial. Int J Antimicrob Agents. 2020;56(1):105949. 10.1016/j.ijantimicag.2020.105949 PMC7102549 32205204

[23] Yuki K, Fujiogi M, Koutsogiannaki S. COVID-19 pathophysiology: A review. Clin Immunol. 2020.;215:108427. 10.1016/j.clim.2020.108427 PMC7169933 32325252

[24] Özdemir Ö, Erkun O (2020). Solving puzzle of the immunopathogenesis for management of COVID-19 disease. MOJ Immunol.

[25] Acosta MA, Singer BD. Pathogenesis of COVID-19-induced ARDS: implications for an ageing population. Eur Respir J. 2020;56(3). 10.1183/13993003.02049-2020 PMC7397945 32747391

[26] Wong JJ, Leong JY, Lee JH, Albani S, Yeo JG. Insights into the immuno-pathogenesis of acute respiratory distress syndrome. Ann Transl Med. 2019;7(19). 10.21037/atm.2019.09.28 PMC6828790 31728357

[27] Pierrakos C, Karanikolas M, Scolletta S, Karamouzos V, Velissaris D (2012). Acute respiratory distress syndrome: pathophysiology and therapeutic options. J Clin Med Res.

[28] Kumar A, Prasoon P, Sekhawat PS, Pareek V, Faiq MA, Kumari C, Narayan RK, Kulandhasamy M, Kant K. Pathogenesis guided therapeutic management of COVID-19: an immunological perspective. Int Rev Immunol. 2021;40(1-2):54–71. 10.1080/08830185.2020.1840566 33111578

[29] Li GG, Cao YH, Run Y, Xu RX, Zheng ZD (2016). Inhibition of CD 8+ T cells and elimination of myeloid cells by CD 4+ Foxp3− T regulatory type 1 cells in acute respiratory distress syndrome. Clin Exp Pharmacol Physiol.

[30] Shimizu Y (2020). Understanding the immunopathogenesis of COVID-19: its implication for therapeutic strategy. World J Clin Cases.

[31] Cao X (2020). COVID-19: immunopathology and its implications for therapy. Nat Rev Immunol.

[32] Hojyo S, Uchida M, Tanaka K, Hasebe R, Tanaka Y, Murakami M (2020). How COVID-19 induces cytokine storm with high mortality. Inflammation Regeneration.

[33] Dadashzadeh N, Farshid S, Valizadeh R, Nanbakhsh M, Rahimi MM. Acute respiratory distress syndrome in COVID-19. Immunopathologia Persa. 2020;6(2):e16-.

[34] Organization WH. Clinical management of severe acute respiratory infection when novel coronavirus ( nCoV) infection is suspected: interim guidance, 25 January 2020: World Health Organization; 2020.

[35] Marini JJ, Gattinoni L. Management of COVID-19 respiratory distress. Jama. 2020;323(22):2329–30. 10.1001/jama.2020.6825 32329799

[36] Messerole E, Peine P, Wittkopp S, Marini JJ, Albert RK (2002). The pragmatics of prone positioning. Am J Respir Crit Care Med.

[37] Sud S, Friedrich JO, Taccone P, Polli F, Adhikari NK, Latini R (2010). Prone ventilation reduces mortality in patients with acute respiratory failure and severe hypoxemia: systematic review and meta-analysis. Intensive Care Med.

[38] COVID-19 treatment guidelines panel. Coronavirus disease 2019 (COVID-19) treatment Guidelinest. https://www.covid19treatmentguidelines.nih.gov/. june25,2020.

[39] Horie S, McNicholas B, Rezoagli E, Pham T, Curley G, McAuley D, O’Kane C, Nichol A, Dos Santos C, Rocco PR, Bellani G. Emerging pharmacological therapies for ARDS: COVID-19 and beyond. Intensive Care Med. 2020;46(12):2265–83. 10.1007/s00134-020-06141-z PMC7352097 32654006

[40] Wang M, Cao R, Zhang L, Yang X, Liu J, Xu M (2020). Remdesivir and chloroquine effectively inhibit the recently emerged novel coronavirus (2019-nCoV) in vitro. Cell Res.

[41] Furuta Y, Komeno T, Nakamura T (2017). Favipiravir (T-705), a broad spectrum inhibitor of viral RNA polymerase. Proceed Japan Acad Ser B.

[42] Cao B, Wang Y, Wen D, Liu W, Wang J, Fan G, Ruan L, Song B, Cai Y, Wei M, Li X. A trial of lopinavir–ritonavir in adults hospitalized with severe Covid-19. N Engl J Med. 2020. 10.1056/NEJMoa2001282 PMC7121492 32187464

[43] Van den Borne B, Dijkmans B, De Rooij H, Le Cessie S, Verweij C (1997). Chloroquine and hydroxychloroquine equally affect tumor necrosis factor-alpha, interleukin 6, and interferon-gamma production by peripheral blood mononuclear cells. J Rheumatol.

[44] Villar J, Ferrando C, Martínez D, Ambrós A, Muñoz T, Soler JA (2020). Dexamethasone treatment for the acute respiratory distress syndrome: a multicentre, randomised controlled trial. Lancet Respir Med.

[45] Glucocorticoid Therapy for COVID-19 Critically Ill Patients With Severe Acute Respiratory Failure. https://ClinicalTrials.gov/show/NCT04244591.

[46] Xu X, Han M, Li T, Sun W, Wang D, Fu B (2020). Effective treatment of severe COVID-19 patients with tocilizumab. Proc Natl Acad Sci.

[47] Richardson P, Griffin I, Tucker C, Smith D, Oechsle O, Phelan A (2020). Baricitinib as potential treatment for 2019-nCoV acute respiratory disease. Lancet (London, England).

[48] Neubauer A, Wiesmann T, Vogelmeier CF, Mack E, Skevaki C, Gaik C (2020). Ruxolitinib for the treatment of SARS-CoV-2 induced acute respiratory distress syndrome (ARDS). Leukemia..

[49] Tang N, Bai H, Chen X, Gong J, Li D, Sun Z (2020). Anticoagulant treatment is associated with decreased mortality in severe coronavirus disease 2019 patients with coagulopathy. J Thromb Haemost.

[50] Moore HB, Barrett CD, Moore EE, McIntyre RC, Moore PK, Talmor DS (2020). Is there a role for tissue plasminogen activator as a novel treatment for refractory COVID-19 associated acute respiratory distress syndrome?. J Trauma Acute Care Surg.

[51] Seifirad S (2020). Pirfenidone: a novel hypothetical treatment for COVID-19. Med Hypotheses.

[52] Nangaku M, Farag YM, deGoma E, Luo W, Vargo D, Khawaja Z. Vadadustat, an oral hypoxia-inducible factor prolyl hydroxylase inhibitor, for treatment of anemia of chronic kidney disease: two randomized Phase 2 trials in Japanese patients. Nephrol Dial Transplant. 2021;36(7):1244–52. 10.1093/ndt/gfaa060 32719868

[53] Warren TK, Jordan R, Lo MK, Ray AS, Mackman RL, Soloveva V (2016). Therapeutic efficacy of the small molecule GS-5734 against Ebola virus in rhesus monkeys. Nature..

[54] Hillaker E, Belfer JJ, Bondici A, Murad H, Dumkow LE. Delayed initiation of remdesivir in a COVID‐19‐positive patient. Pharmacother: J Hum Pharmacol and Drug Ther. 2020;40(6):592–8. 10.1002/phar.2403 PMC7262085 32281114

[55] Piscoya A, Ng-Sueng LF, Parra del Riego A, Cerna-Viacava R, Pasupuleti V, Roman YM (2020). Efficacy and harms of remdesivir for the treatment of COVID-19: a systematic review and meta-analysis. PLoS One.

[56] Wang Y, Zhang D, Du G, Du R, Zhao J, Jin Y, Fu S, Gao L, Cheng Z, Lu Q, Hu Y. Remdesivir in adults with severe COVID-19: a randomised, double-blind, placebo-controlled, multicentre trial. Lancet. 2020;395(10236):1569–78. 10.1016/S0140-6736(20)31022-9 PMC7190303 32423584

[57] Beigel JH, Tomashek KM, Dodd LE, Mehta AK, Zingman BS, Kalil AC, Hohmann E, Chu HY, Luetkemeyer A, Kline S, Lopez de Castilla D. Remdesivir for the treatment of Covid-19. N Engl J Med. 2020;383(19):1813–26. 10.1056/NEJMoa2007764 PMC7262788 32445440

[58] Takahashi H, Iwasaki Y, Watanabe T, Ichinose N, Okada Y, Oiwa A (2020). Case studies of SARS-CoV-2 treated with favipiravir among patients in critical or severe condition. Int J Infect Dis.

[59] Puskarich MA, Cummins NW, Ingraham NE, Wacker DA, Reilkoff RA, Driver BE (2021). A multi-center phase II randomized clinical trial of losartan on symptomatic outpatients with COVID-19. EClinicalMedicine..

[60] Zhou D, Dai SM, Tong Q. COVID-19: a recommendation to examine the effect of hydroxychloroquine in preventing infection and progression. J Antimicrob Chemother. 2020;75(7):1667–70. 10.1093/jac/dkaa114 PMC7184499 32196083

[61] Yan Y, Zou Z, Sun Y, Li X, Xu K-F, Wei Y (2013). Anti-malaria drug chloroquine is highly effective in treating avian influenza a H5N1 virus infection in an animal model. Cell Res.

[62] Réa-Neto Á, Bernardelli RS, Câmara BMD, Reese FB, Queiroga MVO, Oliveira MC (2021). An open-label randomized controlled trial evaluating the efficacy of chloroquine/hydroxychloroquine in severe COVID-19 patients. Sci Rep.

[63] Eze P, Mezue KN, Nduka CU, Obianyo I, Egbuche O (2021). Efficacy and safety of chloroquine and hydroxychloroquine for treatment of COVID-19 patients-a systematic review and meta-analysis of randomized controlled trials. Am J Cardiovasc Dis.

[64] Saghir SA, AlGabri NA, Alagawany MM, Attia YA, Alyileili SR, Elnesr SS (2021). Chloroquine and hydroxychloroquine for the prevention and treatment of COVID-19: a fiction, hope or hype? An updated review. Ther Clin Risk Manag.

[65] COVID MED Trial - Comparison Of Therapeutics for Hospitalized Patients Infected With COVID-19. https://ClinicalTrials.gov/show/NCT04328012.

[66] Nouri-Vaskeh M, Kalami N, Zand R, Soroureddin Z, Varshochi M, Ansarin K (2021). Comparison of losartan and amlodipine effects on the outcomes of patient with COVID-19 and primary hypertension: a randomised clinical trial. Int J Clin Pract.

[67] Bolotova O, Yoo J, Chaudhri I, Marcos LA, Sahib H, Koraishy FM (2020). Safety, tolerability, and outcomes of losartan use in patients hospitalized with SARS-CoV-2 infection: a feasibility study. PLoS One.

[68] Bengtson CD, Montgomery RN, Nazir U, Satterwhite L, Kim MD, Bahr NC (2021). An open label trial to assess safety of losartan for treating worsening respiratory illness in COVID-19. Front Med.

[69] Halpin DM, Singh D, Hadfield RM. Inhaled corticosteroids and COVID-19: a systematic review and clinical perspective. Eur Respir J. 2020;55(5). 10.1183/13993003.01009-2020 PMC7236828 32341100

[70] Russell B, Moss C, Rigg A, Van Hemelrijck M. COVID-19 and treatment with NSAIDs and corticosteroids: should we be limiting their use in the clinical setting?. E Cancer Med Sci. 2020;14. 10.3332/ecancer.2020.1023 PMC7105332 32256706

[71] Alzghari SK, Acuña VS (2020). Supportive treatment with tocilizumab for COVID-19: a systematic review. J Clin Virol.

[72] Feldmann M, Maini RN, Woody JN, Holgate ST, Winter G, Rowland M (2020). Trials of anti-tumour necrosis factor therapy for COVID-19 are urgently needed. Lancet.

[73] So C, Ro S, Murakami M, Imai R, Jinta T (2020). High-dose, short-term corticosteroids for ARDS caused by COVID-19: a case series. Respirol Case Rep.

[74] van Paassen J, Vos JS, Hoekstra EM, Neumann KM, Boot PC, Arbous SM (2020). Corticosteroid use in COVID-19 patients: a systematic review and meta-analysis on clinical outcomes. Crit Care.

[75] Steinberg K (2006). National Heart, Lung, and Blood Institute acute respiratory distress Syndrime (ARDS) clinical trials network. Efficacy and safety of corticosteroids for persistent acute respiratory distress syndrome. N Engl J Med.

[76] COVID-19-associated ARDS Treated With Dexamethasone: Alliance Covid-19 Brasil III. https://ClinicalTrials.gov/show/NCT04327401.

[77] Tomazini BM, Maia IS, Cavalcanti AB, Berwanger O, Rosa RG, Veiga VC (2020). Effect of dexamethasone on days alive and ventilator-free in patients with moderate or severe acute respiratory distress syndrome and COVID-19: the CoDEX randomized clinical trial. Jama..

[78] Group TR. Dexamethasone in hospitalized patients with Covid-19—preliminary report. N Engl J Med. 2020.

[79] Raju R, Prajith V, Biatris PS (2021). Therapeutic role of corticosteroids in COVID-19: a systematic review of registered clinical trials. Future J Pharm Sci.

[80] Ranjbar K, Moghadami M, Mirahmadizadeh A, Fallahi MJ, Khaloo V, Shahriarirad R (2021). Methylprednisolone or dexamethasone, which one is superior corticosteroid in the treatment of hospitalized COVID-19 patients: a triple-blinded randomized controlled trial. BMC Infect Dis.

[81] Ma S, Xu C, Liu S, Sun X, Li R, Mao M (2021). Efficacy and safety of systematic corticosteroids among severe COVID-19 patients: a systematic review and meta-analysis of randomized controlled trials. Signal Transduction Targeted Ther.

[82] Chaudhuri D, Sasaki K, Karkar A, Sharif S, Lewis K, Mammen MJ, Alexander P, Ye Z, Lozano LE, Munch MW, Perner A. Corticosteroids in COVID-19 and non-COVID-19 ARDS: a systematic review and meta-analysis. Intensive Care Med. 2021;19:71. 10.1007/s00134-021-06394-2 PMC8054852 33876268

[83] Kumakawa Y, Hirano Y, Sueyoshi K, Ishihara T, Kondo Y, Kawasaki T (2020). Late iv steroid treatment for severe COVID-19-induced acute respiratory distress syndrome: a case report. Acute Med Surg.

[84] Chan MC, Kuok DI, Leung CY, Hui KP, Valkenburg SA, Lau EH (2016). Human mesenchymal stromal cells reduce influenza a H5N1-associated acute lung injury in vitro and in vivo. Proc Natl Acad Sci.

[85] Lanzoni G, Linetsky E, Correa D, Messinger Cayetano S, Alvarez RA, Kouroupis D (2021). Umbilical cord mesenchymal stem cells for COVID-19 acute respiratory distress syndrome: a double-blind, phase 1/2a, randomized controlled trial. Stem Cells Transl Med.

[86] Shi L, Huang H, Lu X, Yan X, Jiang X, Xu R (2021). Effect of human umbilical cord-derived mesenchymal stem cells on lung damage in severe COVID-19 patients: a randomized, double-blind, placebo-controlled phase 2 trial. Signal Transduction Targeted Ther.

[87] Liang B, Chen J, Li T, Wu H, Yang W, Li Y, Li J, Yu C, Nie F, Ma Z, Yang M. Clinical remission of a critically ill COVID-19 patient treated by human umbilical cord mesenchymal stem cells: A case report. Medicine. 2020;99(31). 10.1097/MD.0000000000021429 PMC7402800 32756149

[88] Kaye RJ (2020). Overview of stem cell therapy for acute respiratory distress syndrome with focus on COVID 19. Pain Physician.

[89] Wang W, Lei W, Jiang L, Gao S, Hu S, Zhao Z-G (2021). Therapeutic mechanisms of mesenchymal stem cells in acute respiratory distress syndrome reveal potentials for Covid-19 treatment. J Transl Med.

[90] Buonaguro FM, Puzanov I, Ascierto PA. Anti-IL6R role in treatment of COVID-19-related ARDS: Springer; 2020. 10.1186/s12967-020-02333-9 PMC7154570 32290847

[91] Toniati P, Piva S, Cattalini M, Garrafa E, Regola F, Castelli F, Franceschini F, Airò P, Bazzani C, Beindorf EA, Berlendis M. Tocilizumab for the treatment of severe COVID-19 pneumonia with hyperinflammatory syndrome and acute respiratory failure: a single center study of 100 patients in Brescia, Italy. Autoimmun Rev. 2020;19(7):102568. 10.1016/j.autrev.2020.102568 PMC7252115 32376398

[92] Rilinger J, Kern WV, Duerschmied D, Supady A, Bode C, Staudacher DL (2020). A prospective, randomised, double blind placebo-controlled trial to evaluate the efficacy and safety of tocilizumab in patients with severe COVID-19 pneumonia (TOC-COVID): a structured summary of a study protocol for a randomised controlled trial. Trials..

[93] Salvarani C, Dolci G, Massari M, Merlo DF, Cavuto S, Savoldi L (2021). Effect of tocilizumab vs standard care on clinical worsening in patients hospitalized with COVID-19 pneumonia: a randomized clinical trial. JAMA Intern Med.

[94] Pinzon RT, Wijaya VO, Buan RB. Interleukin-6 (IL-6) inhibitors as therapeutic agents for Coronavirus disease 2019 (COVID-19): A Systematic Review and Meta-Analysis. J Infect Public Health. 2021. 10.1016/j.jiph.2021.06.004 PMC8204364 34153723

[95] Chen CX, Wang JJ, Li H, Yuan LT, Gale RP, Liang Y. JAK-inhibitors for coronavirus disease-2019 (COVID-19): a meta-analysis. Leukemia. 2021;1–5. 10.1038/s41375-021-01266-6 PMC8119232 33990684

[96] Capochiani E, Frediani B, Iervasi G, Paolicchi A, Sani S, Roncucci P (2020). Ruxolitinib rapidly reduces acute respiratory distress syndrome in COVID-19 disease. Analysis of Data Collection From RESPIRE Protocol. Front Med.

[97] Ruxolitinib for Treatment of Covid-19 Induced Lung Injury ARDS. https://ClinicalTrials.gov/show/NCT04359290.

[98] Roback JD, Guarner J (2020). Convalescent plasma to treat COVID-19: possibilities and challenges. Jama..

[99] Robbiani DF, Gaebler C, Muecksch F, Lorenzi JC, Wang Z, Cho A, Agudelo M, Barnes CO, Gazumyan A, Finkin S, Hägglöf T. Convergent antibody responses to SARS-CoV-2 in convalescent individuals. Nature. 2020;584(7821):437–42. 10.1038/s41586-020-2456-9 PMC7442695 32555388

[100] Premkumar L, Segovia-Chumbez B, Jadi R, Martinez DR, Raut R, Markmann AJ, Cornaby C, Bartelt L, Weiss S, Park Y, Edwards CE. The receptor-binding domain of the viral spike protein is an immunodominant and highly specific target of antibodies in SARS-CoV-2 patients. Sci Immunol. 2020;5(48):eabc8413. 10.1126/sciimmunol.abc8413 PMC7292505 32527802

[101] Stebbing J, Phelan A, Griffin I, Tucker C, Oechsle O, Smith D (2020). COVID-19: combining antiviral and anti-inflammatory treatments. Lancet Infect Dis.

[102] Pouladzadeh M, Safdarian M, Eshghi P, Abolghasemi H, Sheibani B, Choghakabodi PM, Feghhi A, Boroujerdnia MG, Forouzan A, Far MA, Kaydani GA. A randomized clinical trial evaluating the immunomodulatory effect of convalescent plasma on COVID-19-related cytokine storm. Intern Emerg Med. 2021;10:1–1. 10.1007/s11739-021-02734-8 PMC8035885 33837906

[103] Ray Y, Paul SR, Bandopadhyay P, D’Rozario R, Sarif J, Lahiri A, Bhowmik D, Vasudevan JS, Maurya R, Kanakan A, Sharma S. Clinical and immunological benefits of convalescent plasma therapy in severe COVID-19: insights from a single center open label randomised control trial. medRxiv. 2020.

[104] Shen C, Wang Z, Zhao F, Yang Y, Li J, Yuan J (2020). Treatment of 5 critically ill patients with COVID-19 with convalescent plasma. Jama..

[105] Allahyari A, Seddigh-Shamsi M, Mahmoudi M, Jamehdar SA, Amini M, Mozdourian M, Javidarabshahi Z, Abadi SE, Amini S, Sedaghat A, Emadzadeh M. Efficacy and safety of convalescent plasma therapy in severe COVID-19 patients with acute respiratory distress syndrome. Int Immunopharmacol. 2021;93:107239. 10.1016/j.intimp.2020.107239 PMC7709614 33582019

[106] Lee K-Y (2017). Pneumonia, acute respiratory distress syndrome, and early immune-modulator therapy. Int J Mol Sci.

[107] Gharebaghi N, Nejadrahim R, Mousavi SJ, Sadat-Ebrahimi S-R, Hajizadeh R (2020). The use of intravenous immunoglobulin gamma for the treatment of severe coronavirus disease 2019: a randomized placebo-controlled double-blind clinical trial. BMC Infect Dis.

[108] Shao Z, Feng Y, Zhong L, Xie Q, Lei M, Liu Z (2020). Clinical efficacy of intravenous immunoglobulin therapy in critical ill patients with COVID-19: a multicenter retrospective cohort study. Clin Transl Immunol.

[109] Zhang Y, Cao W, Xiao M, Li Y, Yang Y, Zhao J (2020). Clinical and coagulation characteristics in 7 patients with critical COVID-2019 pneumonia and acro-ischemia. Zhonghua xue ye xue za zhi=. Zhonghua Xueyexue Zazhi.

[110] Nebulized Heparin in Severe Acute Respiratory Syndrome COVID-19. https://ClinicalTrials.gov/show/NCT04530578.

[111] MacLaren R, Stringer KA (2007). Emerging role of anticoagulants and fibrinolytics in the treatment of acute respiratory distress syndrome. Pharmacotherapy: the journal of human pharmacology and drug. Therapy..

[112] Hardaway RM, Williams CH, Marvasti M, Farias M, Tseng A, Pinon I (1990). Prevention of adult respiratory distress syndrome with plasminogen activator in pigs. Crit Care Med.

[113] Stringer KA, Hybertson BM, Cho OJ, Cohen Z, Repine JE (1998). Tissue plasminogen activator (tPA) inhibits interleukin-1 induced acute lung leak. Free Radic Biol Med.

[114] Liu C, Ma Y, Su Z, Zhao R, Zhao X, Nie H-G (2018). Meta-analysis of preclinical studies of fibrinolytic therapy for acute lung injury. Front Immunol.

[115] Hardaway RM, Harke H, Tyroch AH, Williams CH (2001). Treatment of severe acute respiratory distress syndrome: a final report on a phase I study. Am Surg.

[116] Wang J, Hajizadeh N, Moore EE, McIntyre RC, Moore PK, Veress LA (2020). Tissue plasminogen activator (tPA) treatment for COVID-19 associated acute respiratory distress syndrome (ARDS): a case series. J Thromb Haemost.

[117] Fibrinolytic Therapy to Treat ARDS in the Setting of COVID-19 Infection. https://ClinicalTrials.gov/show/NCT04357730.

[118] Impact of Tissue Plasminogen Activator (tPA) Treatment for an Atypical Acute Respiratory Distress Syndrome (COVID-19). https://ClinicalTrials.gov/show/NCT04453371.

[119] Nebulised Rt-PA for ARDS Due to COVID-19. https://ClinicalTrials.gov/show/NCT04356833.

[120] Seifirad S, Alquran L (2020). Commentary: Antifibrotics in COVID-19 lung disease: let us stay focused. Front Med.

[121] Jahani M, Dokaneheifard S, Mansouri K (2020). Hypoxia: a key feature of COVID-19 launching activation of HIF-1 and cytokine storm. J Inflamm.

[122] Chen L, Liu P, Gao H, Sun B, Chao D, Wang F (2004). Inhalation of nitric oxide in the treatment of severe acute respiratory syndrome: a rescue trial in Beijing. Clin Infect Dis.

[123] Kobayashi J, Murata I (2020). Nitric oxide inhalation as an interventional rescue therapy for COVID-19-induced acute respiratory distress syndrome. Ann Intensive Care.

[124] Nicholls JM, Poon LL, Lee KC, Ng WF, Lai ST, Leung CY (2003). Lung pathology of fatal severe acute respiratory syndrome. Lancet.

[125] Nitric Oxide Therapy for COVID-19 Patients With Oxygen Requirement. https://ClinicalTrials.gov/show/NCT04476992.

[126] Lei C, Su B, Dong H, Bellavia A, Di Fenza R, Fakhr BS, Gianni S, Grassi LG, Kacmarek R, Morais CC, Pinciroli R. Protocol of a randomized controlled trial testing inhaled Nitric Oxide in mechanically ventilated patients with severe acute respiratory syndrome in COVID-19 (SARS-CoV-2). medRxiv. 2020.

[127] Nitric Oxide Gas Inhalation in Severe Acute Respiratory Syndrome in COVID-19. https://ClinicalTrials.gov/show/NCT04306393.

[128] Knoll MD, Wonodi C (2021). Oxford–AstraZeneca COVID-19 vaccine efficacy. Lancet.

[129] Forman R, Shah S, Jeurissen P, Jit M, Mossialos E. COVID-19 vaccine challenges: What have we learned so far and what remains to be done?. Health Policy. 2021. 10.1016/j.healthpol.2021.03.013 PMC7997052 33820678

